# Surgical Classification of Endometriosis

**DOI:** 10.1055/s-0042-1755588

**Published:** 2022-09-08

**Authors:** João Nogueira Neto, Mauricio Simões Abrão, Eduardo Schor, Julio Cesar Rosa-e-Silva

**Affiliations:** 1 Department of Gynecology and Obstetrics, Faculdade de Medicina, Universidade Federal do Maranhão, São Luis, MA, Brazil; 2 Department of Gynecology, Beneficência Portuguesa de São Paulo, São Paulo, SP, Brazil; 3 Department of Gynecology and Obstetrics, Faculdade de Medicina, Universidade de São Paulo, São Paulo, SP, Brazil; 4 Department of Gynecology, Escola Paulista de Medicina, Universidade Federal de São Paulo, São Paulo, SP, Brazil; 5 Department of Gynecology and Obstetrics, Faculdade de Medicina, Ribeirão Preto, Universidade de São Paulo, Ribeirão Preto, SP, Brazil

Endometriosis is a chronic, benign, estrogen-dependent and multifactorial gynecological disease that mainly affects women of reproductive age. It can be defined by the presence of tissue that resembles the endometrial gland and/or stroma outside the uterus, predominantly although not exclusively, in the female pelvis. 1 It is estimated that 10% of women of reproductive age have this disease, which represents around 176 million women worldwide, generating direct costs to health systems and indirect costs due to reduced productivity, in addition to physical and psychological suffering secondary to pain and infertility, with consequent loss of quality of life. 2

Given the many difficulties imposed by endometriosis, it has been extensively researched in recent decades. 3 4 Its classification is one of the difficulties faced. A reproducible, easy-to-apply, and well-organized classification system is needed not only to clarify communication between clinicians, but also to standardize the optimal treatment strategy and clinical trials. 2 5

The National Specialized Commission on Endometriosis of the Brazilian Federation of Gynecology and Obstetrics Associations – FEBRASGO analyzed the different forms of classification chosen by the World Endometriosis Society (WES) 5 with the objective to standardize the current classification nationwide for Brazilian services that diagnose and treat this disease.

As a single classification that evaluates all possible manifestations of endometriosis is lacking, four classifications were standardized, among which: the revised classification of the American Society for Reproductive Medicine (rASRM), the ENZIAN classification, the Endometriosis Fertility Index (EFI) and the American Association of Gynecologic Laparoscopists (AAGL) classification. 2 6 7 8 9 10

The World Endometriosis Society (WES) published the first international consensus on the classification of endometriosis using a rigorous methodology in 2017. 5 The lack of a classification comprising all aspects of this disease led to the proposal of a combination of the most relevant classifications that could be used by all professionals working with women with endometriosis, from which surgeons can select the appropriate components and ensure its documentation in patients' records. 5

The initial ASRM Classification proposed a single approach in 1979. 6 The endometriosis stage is derived from a cumulative score according to the location and size of lesions observed during surgery. 2 6 The staging system underwent modifications in 1996 and is currently divided into I (1-5 points, minimal), II (6-15 points, mild), III (16-40 points, moderate) and IV (greater than 40 points, severe).

The advantages of this classification are its global acceptance, being widely used, easy application and the fact of helping patients to easily understand the stage of their disease. 2

Among the disadvantages are differences between histologically diagnosed endometriosis and the stage made by visualization, its low reproducibility, low correlation between symptoms and its staging, not assessing the severity of pain and infertility, and not considering the presence of deep infiltrating endometriosis in areas such as uterosacral ligaments, bladder, vagina and intestine. 2 6 11 12

The ENZIAN classification was introduced in 2005 to determine the extent of deep endometriosis during surgical treatment, complementing the rASRM classification. This classification was already revised in 2010 and 2011 to correct its overlap with the rASRM and make it easier to use. 2 7 In 2021, it was revised again to introduce the evaluation of the forms of peritoneal and ovarian endometriosis, and the assessment of tubal permeability through chromotubation and secondary adhesions. 13 This last review aimed to propose a logical anatomical classification for use by a non-invasive method (magnetic resonance imaging and pelvic ultrasound), preoperatively, enabling a more adequate surgical planning, and intraoperatively, allowing a consistent and clear classification of deep endometriosis. Future studies are needed to assess its clinical validity, accuracy and reproducibility. 2 13

The advantages are that it describes the retroperitoneal structures, can be determined by imaging modality and used for surgical planning, and the location and extent of the disease are associated with the presence and severity of different symptoms such as pain. 2 5 14

The following are among the disadvantages: low level of global acceptance due to its complexity; patients' difficulty in understanding the classification informed given the complexity of stages and insufficient knowledge of pelvic anatomy by lay people; the classification will be imprecise if the surgical approach to deep lesions is performed incompletely or if the imaging study is not confirmed in the surgical procedure; and finally, even if the classification is previously made by imaging modality, there is still no scientific evidence on the usefulness of the classification determined by image, although it has great future potential because of the increasing percentage of patients in clinical follow-up of the disease. 2

Another existing classification, the EFI, aims to develop a Fertility Index in patients with endometriosis, and predict the rate of spontaneous pregnancy in patients with endometriosis undergoing surgical treatment who will not attempt to conceive with assisted reproduction techniques. 8

The EFI system considers historical factors such as age, duration of infertility and previous pregnancies associated with intraoperative findings. The functional score indicates the situation of pelvic organs for a possible future spontaneous pregnancy. Functional scores are determined by the surgeon and range from 0 to 4 points as follows; absent or nonfunctional as 0, severe dysfunction as 1, moderate dysfunction as 2, mild dysfunction as 3, and normal as 4. Not only the minimal functional score, but also other surgical factors such as the rASRM total score and the rASRM endometriosis lesion score are included. Finally, the final EFI score is calculated by adding the scores from the history and surgical findings that range from 0 to 10 points, with 10 indicating the best prognosis and 0 the worst prognosis. 2 8

The EFI system has a clear advantage in predicting the outcome of pregnancy and reflects the possible future pregnancy rate better than the rASRM classification, where a score of 6 or more has better RA results than a score of 5 or less. 2 15 16 This classification has already been validated externally numerous times and seems to be an interesting tool for patients with endometriosis and infertility.

However, the EFI system has the following disadvantages: the classification score does not correlate with pain, as it was not designed for this purpose; as the lowest function score is judged subjectively, the total score may vary by surgeon; it is more complex to use than the rASRM classification and the ENZIAN, as it requires the calculation and sum of scores from several categories. 2 8 We believe it is interesting and useful for the group of patients with endometriosis and infertility and for the purpose of calculating probability of a future pregnancy.

In 2010, the AAGL initiated a project to develop a new classification of endometriosis. 9 Thirty endometriosis specialists were asked to assign scores ranging from 0 to 10 points, based on the pain, infertility, and surgical difficulty of patients with endometriosis. In addition, surgical difficulties were categorized into four levels. 9 The visual analogue scale scores and infertility history of patients were collected before surgery for the validation of the scoring system. In 2012, the AAGL Special Interest Group reported that preliminary results presented at the AAGL meeting in Las Vegas were encouraging and the AAGL classification of endometriosis was found to be related to pain, infertility, and surgical difficulty. 11-16

The next step was to conduct a prospective multicenter study with more than 1,500 patients to validate this information. According to its authors, it still requires adjustments and improvements so that it is globally accepted and applied, as well as further investigations and discussions about this new classification. However, initial evaluations concluded that this classification allows the identification of objective intraoperative findings that reliably discriminate the levels of surgical complexity better than the ASRM staging system, and the severity stage correlates with the symptoms of pain and infertility with the ASRM stage. 10 Another interesting data of this classification is its easy application in the form of an application with the creation of a final version in pdf, which facilitates storage and a copy for patients (https://apps.apple.com/us/app/aagl-endo-classification/id1592383297 or https://play.google.com/store/apps/details?id = br.com.medicinia.aagl&hl = en&gl = US). AAGL, as one of the largest global Medical Societies in the field of Gynecological Surgery, is putting efforts to test the use of the classification even before surgery, by imaging methods.

In conclusion, the search for better care for patients with endometriosis is constant given the great implications that this disease brings to physical, social, sexual, reproductive and psychological health. Special attention to its classification is needed so we can standardize it globally. In this sense, we believe the classification recently proposed by AAGL may have all the necessary requirements for its wide future use.
